# Effectiveness of underwater endoscopic mucosal resection versus conventional endoscopic mucosal resection for 10 to 20 mm colorectal polyps: A protocol of systematic review and meta-analysis

**DOI:** 10.1097/MD.0000000000023041

**Published:** 2020-10-30

**Authors:** Yi Liu, Min Shi, Jun Ren, Xiao-li Zhou, Song Liu

**Affiliations:** a Digestive Endoscopy Center; b Second Ward of Gastrointestinal Surgery, Wuhan NO.1 Hospital, Wuhan, China.

**Keywords:** underwater endoscopic mucosal resection, conventional endoscopic mucosal resection, colorectal polyp, protocol, systematic review

## Abstract

**Background:**

Endoscopic mucosal resection (EMR) is a standard method commonly for removing 10 to 20 mm colorectal polyps. While the incidence of residual or recurrent after conventional EMR is remarkably high. Underwater endoscopic mucosal resection (UEMR) as an alternative technique to conventional EMR for removing colorectal polyps has high adenoma detection and complete resection rates, improves patient comfort, decreases sedation needs, eliminates the risks associated with submucosal injection, and reduces snare and diathermy-induced mucosal injury. We will conduct a comprehensive systematic review and meta-analysis to compare the effectiveness of these two therapies in the management of 10 to 20 mm colorectal polyps.

**Methods:**

PubMed, Embase, Cochrane Library, Web of Science, China National Knowledge Infrastructure, China Science and Technology Journal Database and Chinese Biomedical Literature Database will be searched from inception of databases to November 2020 without language limitation. Two reviewers will independently conduct article selection, data collection, and assessment of risk of bias. Any disagreement will be resolved by discussion with the third reviewer. Review Manager Software 5.3 will be used for meta-analysis. The Cochrane risk of bias tool will be used to assess the risk of bias.

**Results:**

This study will provide a systematic synthesis of current published data to compare the effectiveness of UEMR and conventional EMR for 10 to 20 mm colorectal polyps.

**Conclusions:**

This systematic review and meta-analysis will provide clinical evidence as to whether UEMR is more effective and safer than conventional EMR for 10 to 20 mm colorectal polyps.

**Study registration number:**

INPLASY2020100006.

## 1 Introduction

Colorectal cancer (CRC) is one of the common malignant tumors and remains the leading cause of cancer mortality worldwide.^[1,2]^ Early polypectomy of colorectal polyps has been reported to reduce CRC-related mortality.^[3]^ Endoscopic mucosal resection (EMR) is a standard method commonly for removing 10 to 20 mm colorectal polyps.^[4]^ EMR involves fluid injection into the submucosa for creation of a cushion that separates superficial lesions from the underlying muscularis propria to decrease the risk of full-thickness colonic perforation, facilitate entrapment within a snare and militate against transmural thermal injury.^[5–7]^ While conventional EMR has some limitations.^[8–12]^ Submucosal injection may displace the polyp into a less accessible location and constrict the lumen, making it more difficult to access the lesion.^[8,9]^ Incidence of residual or recurrent after conventional EMR is remarkably high.^[10]^ It mandates subsequent more frequent surveillance colonoscopy and increases financial burden for patients.^[11,12]^

In recent years, underwater endoscopic mucosal resection (UEMR) has emerged as an alternative technique to conventional EMR for removing colorectal polyps.^[13]^ Water infusion is used, and submucosal injection is not necessary. Water has a focus and magnification effect to improve diagnostic yield and lesion resolution, and define lesion margins.^[14,15]^ As a heat sink, intraluminal water protects against deep thermal injury, and decreases the risk of thermal injury, post-polypectomy electrocoagulation syndrome, and delayed perforation.^[16]^ Compared with conventional EMR, UEMR has higher adenoma detection and complete resection rates, improves patient comfort, decreases sedation needs, eliminates the risks associated with submucosal injection, and reduces snare and diathermy-induced mucosal injury.^[17–21]^

Up to now, no systematic review or meta-analysis has been performed to compare the effectiveness of UEMR and conventional EMR for 10 to 20 mm colorectal polyps. Therefore, we will conduct a comprehensive systematic review and meta-analysis to compare the effectiveness of these two therapies in the management of 10 to 20 mm colorectal polyps.

## 2 Methods

### 2.1 Study registration

This study has been registered on INPLASY (INPLASY2020100006). This meta-analysis will be performed following Preferred Reporting Items for Systematic Reviews and Meta-Analyses (PRISMA) statement checklist.^[22]^

### 2.2 Eligibility criteria for study selection

#### 2.2.1 Types of studies

All randomized controlled trials (RCTs) comparing the effectiveness of UEMR and conventional EMR for 10 to 20 mm colorectal polyps will be included without language limitation. Case reports, animal experiments and reviews will be excluded.

#### 2.2.2 Types of participants

Participants diagnosed with 10 to 20 mm colorectal polyps will be included without restrictions of nationality, age, gender, and race.

#### 2.2.3 Types of interventions

In the treatment group, patients were given UEMR. It the control group, patients were given conventional EMR.

#### 2.2.4 Types of outcomes

Complete resection rate, residual polyp rate and recurrence rate will be designated as the primary outcome. Secondary outcomes will include procedure time in minutes and the incidence of adverse events (such as immediate bleeding, delayed bleeding, post-polypectomy electrocoagulation syndrome, and delayed perforation).

### 2.3 Search strategy

PubMed, Embase, Cochrane Library, Web of Science, China National Knowledge Infrastructure, China Science and Technology Journal Database and Chinese Biomedical Literature Database will be searched from inception of databases to November 2020 without language limitation. The detailed search strategy for PubMed is shown in Table 1. The similar search strategies will be used for other electronic databases.

**Table 1:**
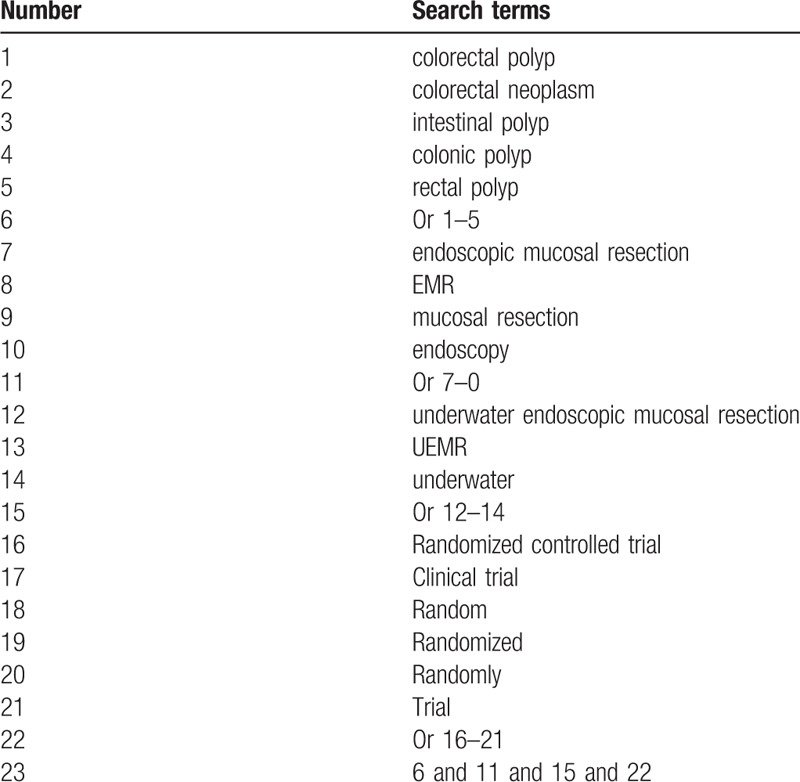
Search strategy of PubMed.

### 2.4 Selection of studies

All the searched articles will be exported to EndNote 7.0 (Thomas Reuters, CA) and duplicates will be excluded by software. Two reviewers will independently scan titles and abstracts to eliminate all irrelevant records. Then, the remaining records will be read by full texts in further assessing the inclusion of the study. Any disagreement about the selection of studies will be resolved by discussion with the third reviewer. A PRISMA flowchart will be designed to describe the details of selection process.

### 2.5 Data extraction and management

After selection, two reviewers will independently conduct data extraction. Any disagreement will be resolved by discussion with the third reviewer. The general information will be extracted, including first author's name, year of publication, title of journal, study design, patient information, experimental intervention, control intervention, and outcomes. If the trials have more than two groups, we only extract the interest-reported information and data. If some important information is missing, we will contact original authors by email to request detailed information about the research.

### 2.6 Assessment of risk of bias

The Cochrane risk of bias assessment tool will be used to assess the risk of bias of the selected studies. Seven items such as random sequence generation, allocation concealment, blinding of participants and personnel, blinding of outcome assessment, incomplete outcome data, selective reporting, and other bias will be assessed by two reviews independently. A bias value of “high,” “unclear,” or “low” was given for each item. The rating results will be cross-checked and the difference will be solved by the third reviewer.

### 2.7 Data synthesis and analysis

#### 2.7.1 Data synthesis

Review Manager Software 5.3 will be used for data synthesis. Risk ratio will be used for dichotomous outcomes with 95% confidence interval. Continuous outcomes will be presented as mean difference or standardized mean difference with 95% confidence interval. The random effects model or fixed effects model will be selected according to the *I*^2^ value. Heterogeneity will be examined using the *I*^2^ test. The *I*^2^ value >50% means significant heterogeneity, and the random effects model will be used. Otherwise, the *I*^2^ value ≤50% means minor heterogeneity, and the fixed effects model will be utilized. If significant heterogeneity still exists after subgroup analysis, meta-analysis will not be pooled, and descriptive summary will be reported.

#### 2.7.2 Subgroup analysis

Subgroup analysis will be performed to check the potential heterogeneity and inconsistency based on the different participant characteristics and outcome indicators.

#### 2.7.3 Sensitivity analysis

Sensitivity analysis will be applied to check the robustness and reliability of pooled results. We will perform meta-analysis again after eliminating studies in low quality and will apply different statistical methods.

#### 2.7.4 Reporting bias

Publication bias will be assessed with funnel plot and Egger regression analysis if sufficient trials (≥10 trials) are included.^[23,24]^

### 2.8 Ethics and dissemination

Ethical approval is not necessary because this study is based on literature analysis. The results of this study will be published in a peer-reviewed journal.

## 3 Discussion

To our knowledge, this is the first systematic review and meta-analysis to conduct a comprehensive literature search and provide a systematic synthesis of current published data to compare the effectiveness of UEMR and conventional EMR for 10 to 20 mm colorectal polyps. We will search seven electronic literature databases to avoid missing any potential eligible studies, and apply rigorous methodology to examine studies reporting UEMR versus conventional EMR for 10 to 20 mm colorectal polyps. We believe that this systematic review and meta-analysis will provide clinical evidence for the effectiveness of UEMR treatment for colorectal polyp and inform our understanding of the value of UEMR in lowering residual polyp rate and recurrence rate, shortening procedure time and reducing incidence of adverse events.

## Author contributions

**Conceptualization:** Yi Liu, Min Shi, Song Liu.

**Data curation:** Jun Ren, Xiao-li Zhou.

**Formal analysis:** Yi Liu, Min Shi, Jun Ren, Xiao-li Zhou, Song Liu.

**Funding acquisition:** Xiao-li Zhou.

**Investigation:** Jun Ren, Xiao-li Zhou.

**Methodology:** Yi Liu, Min Shi, Song Liu.

**Project administration:** Song Liu.

**Resources:** Xiao-li Zhou, Song Liu.

**Software:** Yi Liu, Min Shi.

**Supervision:** Yi Liu, Min Shi, Jun Ren, Xiao-li Zhou.

**Validation:** Song Liu.

**Visualization:** Yi Liu, Min Shi.

**Writing – original draft:** Yi Liu, Min Shi.

**Writing – review & editing:** Yi Liu, Min Shi, Jun Ren, Xiao-li Zhou, Song Liu.
